# Serious infection risk in rheumatoid arthritis compared with non-inflammatory rheumatic and musculoskeletal diseases: a US national cohort study

**DOI:** 10.1136/rmdopen-2019-000935

**Published:** 2019-06-09

**Authors:** Bella Mehta, Sofia Pedro, Gulsen Ozen, Andre Kalil, Frederick Wolfe, Ted Mikuls, Kaleb Michaud

**Affiliations:** 1 Department of Medicine, Hospital for Special Surgery, New York, New York, USA; 2 Forward, The National Databank for Rheumatic Diseases, Wichita, Kansas, USA; 3 Department of Medicine, University of Nebraska Medical Center, Omaha, Nebraska, USA

**Keywords:** rheumatoid arthritis, serious infection, cohort study, non-inflammatory rheumatic disease

## Abstract

**Objectives:**

To identify serious infection (SI) risk by aetiology and site in patients with rheumatoid arthritis (RA) compared with those with non-inflammatory rheumatic and musculoskeletal diseases (NIRMD).

**Methods:**

Patients participating in FORWARD from 2001 to 2016 were assessed for SIs; defined by infections requiring hospitalisation, intravenous antibiotics or followed by death. SIs were categorised by aetiology and site. SI risk was assessed through Cox proportional hazards models. Best models were selected using machine learning Least Absolute Shrinkage and Selection Operator (LASSO) methodology.

**Results:**

Among 20 361 patients with RA and 6176 patients with NIRMD, 1600 and 276 first SIs were identified, respectively. Incidence of SIs was higher in RA compared with NIRMD (IRR = 1.5; 95% CI 1.2 to 1.5). The risk persisted after adjusting using the LASSO model (HR 1.7; 95% CI 1.5 to 1.8), but attenuated when additionally adjusted for glucocorticoid use (HR 1.3; 95% CI 1.2 to 1.5). SI risk was significantly higher in RA versus NIRMD for bacterial infections as well as for respiratory, skin, bone, joint, bloodstream infections and sepsis irrespective of glucocorticoid use. Compared with NIRMD, SI risk was significantly increased in patients with RA who were in moderate and high disease activity but was similar to those in low disease activity/remission (p trend < 0.001).

**Conclusions:**

The risk of all SIs, particularly bacterial, respiratory, bloodstream, sepsis, skin, bone and joint infections are significantly increased in patients with RA compared with patients with NIRMD. This infection risk appears to be greatest in those with higher RA disease activity.

**Key messages**

**What is already known about this subject?**

- Patients with rheumatoid arthritis (RA) are at an increased risk of serious infection (SI) compared with patients without RA.

**What does this study add?**

- This is the first study to assess SI risk in patients with RA compared with patients with non-inflammatory rheumatic and musculoskeletal diseases (NIRMDs) using a nationwide longitudinally followed cohort of patients after introduction of biologics.
- We used the least absolute shrinkage and selection operator (LASSO) technique, which is a modern selection method for confounder selection which does not rely on arbitrary thresholds.

**How might this impact on clinical practice?**

- By identifying SI risk by aetiology and sites of SIs, and how this risk might differ based on underlying RA disease activity we can improve treatment for patients with RA who are at a higher risk of SIs.
- Incidence of SI in patients with RA was 50% higher than those with NIRMD and persisted after LASSO multivariable adjustment (HR 1.7; 95% CI 1.5 to 1.9) without glucocorticoid and (HR 1.3; 95% CI 1.2 to 1.5) with glucocorticoid use. The risk increased proportionately as RA activity worsened.

## Introduction

Serious infections (SIs) in patients with rheumatoid arthritis (RA) are a perpetual concern. The risk of SIs, and the morbidity and mortality associated with SIs is increased in RA.^1–4^ SIs are one of the leading causes of mortality in RA.^5–7^ The increased risk of SIs in RA may be explained by the immunopathogenesis of the disease itself, comorbid conditions and/or immunosuppressive medications. The increased incidence of community-acquired as well as opportunistic infections have been described in the context of immunosuppression in patients with RA.^8^ Early published reports described the increased risk of SIs and related mortality in RA.^3^ At that point, RA treatment was limited and difficult to treat.

The use of more aggressive treatment strategies with cytokine and targeted immune cell blocking agents over the last two decades has increased interest in estimating the risk of SIs in RA. This risk cannot be accurately estimated by clinical trial data, which are typically limited in terms of the number of patients, their external validity and follow-up duration. Although often providing longer follow-up, biologic registries have an inherent channelling bias and therefore may not accurately reflect the risk of all RA patient groups.^9 10^ Observational cohorts have focused on comparing SI rates across different treatments or on infections affecting a single site.^11 12^ Moreover, these studies usually lacked important clinical data such as RA disease activity and severity measures,^13–15^ a critical gap as these factors may be independent risk factors for SI.^16^ In everyday practice, clinicians and patients alike would benefit from an improved understanding of SI risk, including differing aetiologies, sites, and how this risk might differ based on underlying RA disease activity. Together, this information would be helpful for risk estimation of SIs in patients with RA. In this study, our objective was to assess SI risk in patients with RA compared with patients with non-inflammatory rheumatic and musculoskeletal diseases (NIRMDs) using a nationwide longitudinally followed cohort in addition to characterising SI risk by aetiology and site of infection. To adjust for confounders, we wanted to use a modern technology for variable selection—least absolute shrinkage and selection operator (LASSO).

## Methods

### Patient data

We analysed data collected prospectively from 2001 to 2016 from FORWARD, The National Databank for Rheumatic Diseases. FORWARD is an ongoing longitudinal observational patient-driven study that collects information from patients with rheumatic diseases through questionnaires completed at 6-month intervals.^17 18^ Participants are recruited through rheumatology clinics throughout USA, and all have physician-provided diagnoses^19^ (online supplementary appendix 1). All patients provided informed written consent to participate. We identified adult patients (age ≥18 years) with RA. The comparator NIRMD group included patients with primary diagnoses of osteoarthritis, back pain syndromes, tendonitis and other periarticular pain syndromes without any inflammatory rheumatic disease.

10.1136/rmdopen-2019-000935.supp1 Supplementary data

### Outcome and follow-up

The primary outcome was an SI defined as an infection that required intravenous antibiotics (inpatient or outpatient), led to hospitalisation or was followed by death. SIs were validated and confirmed using International Classification of Diseases, Ninth Revision (ICD-9) codes from medical records (physician and hospital), and national death records^19^ (online supplementary table 1). A detailed description of the data validation process in Forward is included as online supplementary appendix 1.^19^ Aetiology and site of SIs were obtained if available. Individual time-at-risk began at enrolment or on 1 January 2001, whichever occurred last, and follow-up continued until the first SI, or censoring at death, loss to follow-up or end of study period (31 December 2016).

SI aetiology was classified as bacterial, viral, fungal or unknown. Opportunistic infections were reported separately. Opportunistic infections were defined as SI caused by a microorganism that normally does not cause disease but becomes pathogenic only when the host's immune system is impaired. Opportunistic infections included events related to *Cryptococcus neoformans*, *Herpes simplex*, *Histoplasma capsulatum*, *Listeria monocytogenes, Mycobacterium tuberculosis*, *Pneumocystis jiroveci* (carinii) among others.^20^ Given the increased risk for Herpes zoster in RA overall,^21^ and increased interest given certain medications particularly increase its risk,^22^ we reported SIs due to Herpes zoster separately. SIs by site were classified into respiratory; abdominal; central nervous system; urinary; bloodstream and sepsis; skin, bone and joint; and unknown.

### Covariates

Baseline covariates included age, sex, education, residence (urban vs rural), insurance (Medicare vs others) and annual income, smoking status, body mass index (BMI), disease duration, Rheumatic Disease Comorbidity Index (RDCI: 0 to 9), diabetes, pulmonary disease, history of fractures, Health Assessment Questionnaire (HAQ), pain and patient global scores assessed by Visual Analogue Scale (0–10).^23 24^ As we wanted to assess some comorbidities included in RDCI individually which can influence SI risk such as diabetes, pulmonary disease and fractures, we dropped the points coming from these comorbidities from RDCI (modified RDCI: 0 to 5). Prior infections were collected as self-reported infections at enrolment. Specific vaccinations were defined as present as a binary variable if the patient had Herpes zoster, influenza or pneumonia vaccinations. Disease activity was assessed at 6-monthly intervals by the Patient Activity Score (PAS, 0–10).^25^ Medication information including time-varying use of glucocorticoids (GCs) for all patients, and for patients with RA, conventional synthetic disease-modifying antirheumatic drugs (csDMARDs (hydroxychloroquine, leflunomide, methotrexate and sulfasalazine), biological (b) DMARDs (infliximab, etanercept, adalimumab, certolizumab, golimumab, abatacept, rituximab, tocilizumab, anakinra), and tofacitinib were collected throughout the follow-up.

### Statistical analysis

Baseline characteristics of patients with RA and NIRMD were compared using descriptive statistics. Covariates are described separately for patients who did develop SIs and those who did not. Crude incidence rate (IR) and incidence rate ratios (IRRs) for first SIs in RA versus NIRMD were calculated per 1000 patient-years. Univariable and multivariable Cox proportional hazards regression models were used to estimate the risk of first SIs. The base model was adjusted for age and sex while final models were adjusted for the remainder of the aforementioned covariates (except for DMARDs) and prior self-reported SI before enrolment. Best models were selected using LASSO applied to the Cox proportional hazards models, when analysing time to SI. Model selection was performed with and without GCs given that patients with RA have an increased use of GC. LASSO is a machine learning methodology that maximises the partial likelihood of the regression coefficients subject to a constraint imposed on the sum of the absolute value of all regression coefficients. The constraint was estimated via cross-validation (online supplementary table 2).^26^ LASSO, an automatic procedure allows for adequate control of confounders which the greatest effect size and does not rely on arbitrary thresholds.

Different models for analysing recurrent SI events were also estimated using the Andersen Gill (AG) or Prentice, Williams and Peterson (PWP) model; both are extensions of the Cox regression model. The AG model assumes that all events have equal risk, reducing the problem for analysing time to first infection, time to second, and so on. The PWP, known as the conditional risk set model, stratifies the analysis by failure order, a subject is not at risk of a second event until the first event has occurred and so on.^27^

In sensitivity analyses, patients with RA were stratified according to disease activity states by PAS as remission/low disease activity (PAS ≤3.7), moderate disease activity (PAS >3.7 and<8), and high disease activity (PAS ≥8). We estimated SI risk of patients with RA in each disease activity group compared with patients with NIRMDs (reference) using similar multivariable LASSO models.^25^ This disease activity group was time varying, meaning that a patient could contribute to more than one disease activity state during follow-up. Since patients with NIRMD would not traditionally be prescribed DMARDs, we categorised patients with RA as patients on csDMARDs and patients on bDMARDs or tofacitinib (regardless of concomitant csDMARDs), alone or in combination with GC, and then compared the SI risk in each category with that of NIRMD (reference) after adjusting for disease activity in the multivariable LASSO regression model. These five treatment categories were (1) NIRMD (reference). (2) csDMARD without GC. (3) csDMARDs with GC. (4) bDMARDs without GC. (5) bDMARDs with GC.

The pattern of missing data in this study was missing at random. In order to prevent bias from removing observations due to missing data, all missing demographics and RA severity covariate data were replaced by using multiple imputation by chained equations to create multiple imputed data sets for analyses.^6^ The level of missingness was <7.5%. Treatment such as GC use was analysed as observed, with no imputation (<4% missing) and DMARD treatment was only available for 16 775 patients with RA in the sensitivity analysis (indicated in table 1). Validated infections events were analysed as observed, assuming that those with missing events, had no cases.

**Table 1:** Baseline characteristics of patients with RA and NIRMD by serious infections‡

	Patients without serious infections			Patients with serious infections		
	RA N=18 707	NIRMD N=5895	P values	RA N=1600	NIRMD N=276	P values
Age, years	57.7 (13.8)*	62.5 (13.4)†	**0.01**	66.8 (12.0)*	72 (11.6)†	**< 0.001**
Male, %	19.8 *	19.5	0.511	24.6*	22.1	0.301
White, %	94.2	96.6	**< 0.001**	94.7	95.7	0.504
BMI, kg/m^2^	28.9 (7.1)	29.3 (7.3)†	**< 0.001**	28.4 (7.5)	30.6 (8.5)†	**< 0.001**
Rural, %	26.4 *	23.9 †	**< 0.001**	32.2*	25.3†	**0.023**
Prior infections, %	4.7 *	3.6	**0.001**	11.9*	11.6	0.037
Ever-smokers, %	40.6 *	34.7†	**< 0.001**	50.6*	40.6†	**0.002**
Diabetes, %	8.2*	8.5†	0.557	14.0*	18.1†	0.086
Disease duration, years	13.8 (12.5)*	15.4 (13.4)†	**< 0.001**	19.9 (12.8)*	22 (15.2)†	0.460
Modified RDCI (0 to 5)	1.4 (1.1)*	159 (1.1)†	**< 0.001**	1.7 (1.1)*	1.9 (1.1)†	**< 0.001**
HAQ (0–3)	1.1 (0.7)*	1.1 (0.7)	0.64	1.3 (0.7)*	1.2 (0.7)	0.150
Pain (0–10)	4.2 (2.9)*	4.2 (2.8)†	0.74	4.5 (2.8)*	4.8 (2.9)†	0.910
Patient global (0–10)	3.8 (2.6)*	3.8 (2.5)†	0.78	4.3 (2.5)*	4.2 (2.5)†	0.470
PAS (0–10)	3.9 (2.3)*	^--^	--	4.41 (2.19)*	--	-**-**
*Remission/low activity, %*	47.6*	--		37.9*	--	--
*Moderate activity, %*	49.3*	--		57.8*	--	
*High activity, %*	3.1*	--		4.3*	--	
bDMARDs, % (n=16 775)	35.3*	--		39.3*	--	
Glucocorticoids, %	28.3	4.4	**< 0.001**	47.4	9.29	**< 0.001**

*Statistically significant difference (p<0.05) between RA with and without serious infection.

†Statistically significant difference (p<0.05) between NIRMD with and without serious infection.

‡The values are presented as mean (SD) unless indicated otherwise.

bDMARDs, Biological disease-modifying antirheumatic drugs (includes infliximab, etanercept, adalimumab, certolizumab, golimumab, abatacept, rituximab, tocilizumab, anakinra); BMI, body mass index;HAQ, Health Assessment Questionnaire;NIRMD, non-inflammatory rheumatic and musculoskeletal diseases;PAS, Patient Activity Scale (Remission/low disease activity ≤3 >3.7 and <8, High activity ≤8).7, Moderate activityRA, rheumatoid arthritis;RDCI, Rheumatic Disease Comorbidity Index.

All analyses were performed using STATA V.4.2 (StataCorp, College Station, Texas, USA) and package GLMNET in R V.3.4 for the LASSO selection. All tests were two-sided and were considered statistically significant when p<0.05.

## Results

The study included 20 361 patients with RA and 6176 patients with NIRMD contributing to 81 499 and 20 665 patient-years of observation and 1600 and 276 first SIs, respectively. Baseline characteristics of patients with RA and NIRMD by SI status are presented in table 1. Patients with RA with SI compared with NIRMD with SI were younger (66.8±12.0 vs 72±11.6 years), were more likely to have ever smoked (50.6% vs 40.6%) and have lower BMI (28.4±7.5 vs 30.6±8.5). The majority of patients resided in urban areas, and SIs were more frequent among patients residing in urban areas both in patients with RA and NIRMD than those residing in rural areas.

Crude IRs and IRRs of SI by aetiology and site are shown in table 2. IRRs of all SIs (1.5; 95% CI 1.3 to 1.7), opportunistic SIs (3.0; 95% CI 1.4 to 1.7) and Herpes zoster SIs (2.2; 95% CI −0.9 to 6.2) were higher in RA compared with NIRMD. The most frequent first SIs by aetiology were bacterial (49.3% in RA and 43.8% in NIRMD) and by site were respiratory (56.8% in RA and 57.2% in NIRMD). Herpes zoster SIs occurred in only 3.3% (n=53) of all RA and 2.1% (n=6) of NIRMD SIs. IRs for opportunistic infections and Herpes zoster SIs were two to three times higher in RA compared with NIRMD (online supplementary table 3). Fungal SIs (IRR 5.1; 95% CI 1.3 to 43.3) in RA were significantly more frequent than in NIRMD. For the SIs, only 5.1% of RA and 2.8% of patients with NIRMD had ‘unknown site’ whereas 60.9% of RA and 63.4% of NIRMD had ‘unknown aetiology’ (~80% of these cases were respiratory infections).

**Table 2:** Incidence rates, incidence rate ratios and HRs (95% CI) for serious infections in patients with RA and NIRMD by the infection, aetiology and site

Infection	Number of cases (n)		Incidence rate (1000 patient years)		Incidence rate ratio (unadjusted)†
	RA	NIRMD	RA	NIRMD	
All infections	1600	276	19.6	13.4	**1.5**
			(18.7–20.6)	(11.9–15.0)	**(1.3– 1.7 )**
Opportunistic	85	7	1.0	0.3	**3.0**
			(0.8–1.2)	(0.2–0.7)	**(1.4– 7.7 )**
Herpes zoster	53	6	0.6	0.3	2.2
			(0.5–0.8)	(0.1–0.6)	(0.9–6.2)
By aetiology					
Bacterial	790	121	9.3	5.7	**1.6**
			(8.7–9.9)	(4.8–6.8)	**(1.3– 2.0 )**
Viral	101	17	1.2	0.8	1.5
			(0.9–1.4)	(0.5–1.3)	(0.9–2.6)
Fungal	41	2	0.5	0.1	**5.1**
			(0.3–0.6)	(0.0–0.4)	**(1.3– 43.3 )**
Unknown aetiology	975	175	11.6	8.3	**1.4**
			(10.9–12.3)	(7.1–9.6)	**(1.2– 1.7 )**
By site					
Respiratory	909	158	10.7	7.4	**1.4**
			(10.1–11.5)	(6.4–8.7)	**(1.2– 1.7 )**
Central nervous system	17	3	0.2	0.1	1.4
			(0.1–0.3)	(0.0–0.4)	(0.4–7.5)
Abdominal	80	10	0.9	0.5	**2.0**
			(0.7–1.1)	(0.2–0.9)	**(1.0– 4.3 )**
Urinary	50	16	1.8	0.7	0.8
			(1.5–2.1)	(0.5–1.2)	(0.4–1.5)
Bloodstream including sepsis	340	47	3.9	2.2	**1.8**
			(3.5–4.3)	(1.6–2.9)	**(1.3– 2.5 )**
Skin, bone and joint infections	486	81	5.7	3.8	**1.5**
			(5.2–6.2)	(3.0–4.7)	**(1.2– 1.9 )**
Unknown site	83	8	0.9	0.4	**2.6**
			(0.8–1.2)	(0.2–0.7)	**(1.2– 6.2 )**

*See online supplementary appendix for details in the variables selected for each LASSO (Least Absolute Shrinkage and Selection Operator) model.

†Bold, p<0.05

NIRMD, non-inflammatory rheumatic and musculoskeletal disease; RA, rheumatoid arthritis.

After adjusting for age and sex, the risk of all SIs was increased in RA compared with NIRMD (HR 1.8; 95% CI 1.5 to 2.0) and after multivariable adjustment and LASSO selection (without GC) (HR 1.7; 95% CI 1.5 to 1.9). This risk was attenuated when including GC use in the model; it however, remained statistically significant (HR 1.3; 95% CI 1.2 to 1.5) (table 3;Online supplementary figure S1 and S2). Similarly, the risk of opportunistic SIs was increased in RA versus NIRMD (HR 3.1; 95% CI 1.4 to 6.6) in age-adjusted and sex-adjusted models, and multivariable LASSO model without GC use (HR 2.7; 95% CI 1.3 to 6.0) and attenuated further with GC use (HR 1.7; 95% CI 0.8 to 3.9). By aetiology, in adjusted models, the risk of bacterial infections was significantly higher in patients with RA than NIRMD (HR 1.9; 95% CI 1.5 to 2.3 without GC and HR 1.5; 95% CI 1.2 to 1.8 with GC). Patients with RA had significantly higher risk of respiratory, abdominal, bloodstream, sepsis, skin, bone and joint SIs than patients with NIRMD, after multivariable adjustment (table 3). Current use of GC was the strongest predictor for all (HR 2.2; 95% CI 2.0 to 2.4), opportunistic (3.5; 95% CI 2.2 to 5.4) and Herpes zoster SIs (HR 3.7; 95% CI 2.2 to 6.4) (table 3) and was clearly the factor responsible for the strong attenuation of HR when comparing RA versus NIRMD (table 4). Female sex, diabetes, rural residency, more comorbidities, history of prior SIs and worse HAQ disability scores were also associated with increased SI risk in RA compared with NIRMD, in both models, with or without GC use.

**Table 3:** HRs (95% CIs) for serious infections in patients with RA and NIRMD by the infection, aetiology and site, age and sex adjustment, and using LASSO with GC and without

Infection	Age and sex adjusted model	LASSO model HR (with GC) (95% CI) *	LASSO model HR (without GC) (95% CI) *
All infections	**1.8**	**1.3**	**1.7**
	**(1.5– 2.0 )**	**(1.2– 1.5)**	**(1.5– 1.9)**
Opportunistic	**3.1**	1.7	**2.7**
	**(1.4– 6.6 )**	(0.8–3.9)	**(1.3– 6.0)**
Herpes zoster	**2.9**	1.7	**2.8**
	**(1.2– 6.7 )**	(0.7–4.2)	**(1.2– 6.7 )**
By aetiology			
Bacterial	**1.9**	**1.5**	**1.9**
	**(1.6– 2.3 )**	**(1.2– 1.8 )**	**(1.5– 2.3 )**
Viral	**1.7**	1.2	1.5
	**(1.0– 2.8 )**	(0.7–2.0)	(0.9–2.5)
Fungal	**5.1**	2.7	**4.6**
	**(1.2– 21.0 )**	(0.6–11.7)	**(1.1– 19.5 )**
Unknown aetiology	**1.7**	**1.3**	**1.6**
	**(1.5– 2.0 )**	**(1.0– 1.5 )**	**(1.4– 1.9 )**
By site			
Respiratory	**1.8**	**1.3**	**1.6**
	**(1.5– 2.2 )**	**(1.1– 1.6 )**	**(1.4– 2.0 )**
Central nervous system	**1.4**	1.0	1.5
	**(1.1– 1.6 )**	(0.3–3.7)	(0.4–5.2)
Abdominal	**2.1**	1.5	**2.1**
	**(1.1– 4.1 )**	(0.7–2.9)	**(1.1– 4.1 )**
Urinary	0.9	0.7	0.8
	(0.5–1.6)	(0.4–1.4)	(0.4–1.4)
Bloodstream including sepsis	**2.2**	**1.7**	**2.2**
	**(1.6– 2.9 )**	**(1.2– 2.4 )**	**(1.6– 3.0 )**
Skin, bone and joint infections	**1.7**	**1.4**	**1.7**
	**(1.3– 2.1 )**	**(1.1– 1.8 )**	**(1.4– 2.2 )**
Unknown site	**2.9**	1.6	**2.6**
	**(1.4– 6.0 )**	(0.7–3.3)	**(1.2– 5.3 )**

*Bold, p<0.05

GC, glucocorticoid; LASSO, least absolute shrinkage and selection operator; NIRMD, non-inflammatory rheumatic and musculoskeletal diseases; RA, rheumatoid arthritis.

**Table 4:** Factors associated with serious infection risk in patients with RA and NIRMD using the best models selected by LASSO, excluding and including GC*

Time to SI	All HR (95% CI)		Opportunistic HR (95% CI)		Herpes zoster HR (95% CI)	
	Excluding GC	Including GC	Excluding GC	Including GC	Excluding GC	Including GC
RA versus NIRMD	**1.7** **(1.5– 1.9 )**	**1.3** **(1.2 –1.5 )**	**2.7** **(1.3– 6.0 )**	1.7 (0.8–3.9)	**2.8** **(1.2– 6.7 )**	1.7 (0.7–4.2)
White race	**1.3** **(1.1– 1.7 )**	**1.3** **(1.1 –1.7 )**			**1.2** **(0.4– 3.8 )**	
Age (years)	**1.0** **(1.0– 1.0 )**	**1.0** **(1.0– 1.0 )**	**1.0** **(1.0– 1.0 )**	**1.0** **(1.0– 1.0 )**	**1.0** **(1.0– 1.1 )**	**1.0** **(1.0– 1.1 )**
Female sex	**1.3** **(1.2– 1.4 )**	**1.2** **(1.1– 1.4 )**	1.5 (0.9–2.5)	1.5 (0.9–2.5)		
Disease duration	**1.0** **(1.0– 1.0 )**	**1.0** **(1.0– 1.0 )**	**1.0** **(1.0– 1.0 )**	**1.0** **(1.0– 1.0 )**		
Rural (vs urban)	**1.1** **(1.0– 1.2 )**	**1.1** **(1.0– 1.2 )**				
Prior infections	**1.4** **(1.2– 1.7 )**	**1.4** **(1.2– 1.6 )**	1.3 (0.7–2.6)		1.9 (0.9–4.1)	1.9 (0.9–4.1)
Ever smoker	**1.2** **(1.1– 1.3 )**	**1.2** **(1.1– 1.3 )**	0.7 (0.4–1.1)	0.6 (0.4–1.0)	0.6 (0.4–1.1)	0.6 (0.3–1.0)
Modified RDCI (0 to 5)	**1.1** **(1.1– 1.2 )**	**1.1** **(1.1– 1.2 )**			**1.3** **(1.0– 1.6 )**	
HAQ disability	**1.4** **(1.3– 1.5 )**	**1.3** **(1.2– 1.4 )**		0.9 (0.7–1.4)	**1.7** **(1.2– 2.4 )**	**1.6** **(1.1– 2.3 )**
Pain scale	**1.0** **(1.0– 1.1 )**	**1.0** **(1.0– 1.0 )**		1.0 (0.9–1.1)		
Educational level	1.0 (0.9–1.0)	**1.0** **(0.9 – 1.0)**	1.0 (0.9–1.1)	1.0 (0.9–1.1)		
Diabetes	**1.5** **(1.3– 1.7 )**	**1.5** **(1.3– 1.7 )**	0.6 (0.3–1.3)	0.6 (0.3–1.4)	0.3 (0.1–1.1)	0.3 (0.1–1.2)
Vaccinations	**0.7** **(0.7– 0.8 )**	**0.7** **(0.7 – 0.8)**	1.1 (0.7–1.7)	1.1 (0.7–1.8)	0.7 (0.4–1.2)	0.7 (0.4–1.3)
Pulmonary disease	**1.7** **(1.5– 1.8 )**	**1.6** **(1.5– 1.8 )**	**3.3** **(2.1– 5.1 )**	**3.2** **(2.1– 5.0 )**		
History of fracture	**1.4** **(1.3– 1.6 )**	**1.4** **(1.2– 1.5 )**	0.9 (0.6–1.5)	0.9 (0.5–1.5)	**2.8** **(1.2– 6.7 )**	
Current use of glucocorticoid	**1.7** **(1.5– 1.9 )**	**2.2** **(2.0– 2.4 )**	**2.7** **(1.3– 6.0 )**	**3.5** **(2.2– 5.4 )**	**2.8** **(1.2– 6.7 )**	**3.7** **(2.2– 6.4 )**

*Bold, p<0.05

GC, glucocorticoids;HAQ, Health Assessment Questionnaire; LASSO, least absolute shrinkage and selection operator; NIRMD, non-inflammatory rheumatic and musculoskeletal diseases;RA, rheumatoid arthritis;RDCI, Rheumatic Disease Comorbidity Index; SI, serious infection.

When analysing time to recurrent SI using the AG model, the HR for all infections was 1.7 (95% CI 1.5 to 1.9) without GC use and 1.3 (95% CI 1.2 to 1.5) with GC use, which was identical to time to the first SIs described in table 3 (HR 1.7; 95% CI 1.5 to 1.9 without; HR 1.3; 95% CI 1.2 to 1.5 with GC). However, when using a model with a different assumption (PWP), conditional on the prior infections, the risk of recurrent SI was only slightly increased, 1.1 (95% CI 1.0 to 1.2) p=0.09 with GC use and 1.1 (95% CI 1.0 to 1.3) p=0.04 without GC use, which indicated that the results from the AG model were predominantly driven by the first infection (results not shown by aetiology or site).

**Figure 1:**
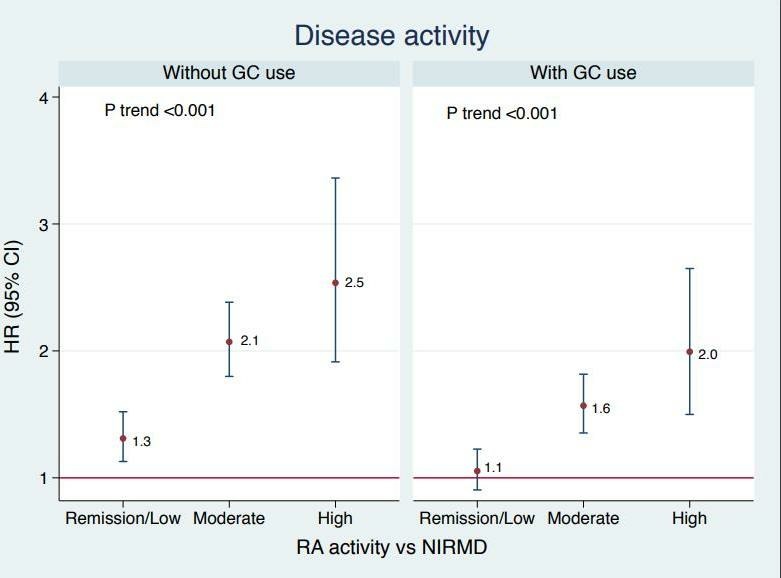
Patients with RA in remission/low, moderate and high disease activity according to the PAS (Patient activity Score) scale on the x axis. HRs of all serious infections on y axis (adjusting for age, sex, race, residency (urban vs rural), RA disease duration, diabetes, pulmonary disease, fractures, prior infection, smoking status, Modified Rheumatic Disease Comorbidity Index, education level and vaccination status) per LASSO selection. GC, glucocorticoids; LASSO, least absolute shrinkage and selection operator; NIRMD, non-inflammatory rheumatic and musculoskeletal diseases; RA, rheumatoid arthritis.

The IR of SI in patients with RA when categorised by disease activity, is lower in remission/low disease activity (IR 13.4; 95% CI 12.4 to 14.6) than in patients with moderate (IR 26.7; 95% CI 25.1 to 28.5) and high disease activity (IR 41.3; 95% CI 32.5 to 52.4) (online supplementary table 4). In multivariable assessments of the relationship between RA disease activity and SI risk using selected LASSO models, we observed a significant uptrend in SI risk in RA as disease activity increased compared with NIRMD irrespective of GC use (p-trend <0.001). Patients with RA who were in remission/low disease activity state had a slightly higher SI risk compared with NIRMD (HR 1.1; 95% CI 0.9 to 1.2 adjusted with GC; HR 1.3; 95% CI 1.1 to 1.5 adjusted without GC), whereas those who were in moderate (HR 1.6; 95% CI 1.4 to 1.8 adjusted with GC; HR 2.1; 95% CI 1.8 to 2.4 adjusted without GC) and high (HR 2.0; 95% CI 1.5 to 2.6 adjusted with GC; HR 2.5; 95% CI 1.9 to 3.4 adjusted without GC) disease activity states had a proportionately increased SI risk compared with NIRMD (figure 1A, B).

**Figure 2:**
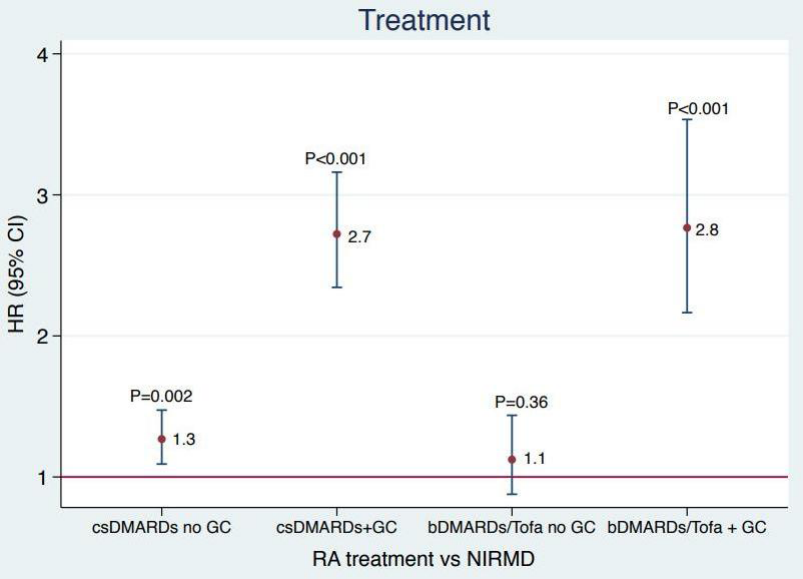
Patients with RA on csDMARDs with and without GC, bDMARDs/tofacitinib with and without GC on the x axis. HR of all seriousinfections on y axis (adjusting for age, sex, race, Health Assessment Questionnaire (HAQ), pain scale, Modified Rheumatic Disease Comorbidity Index, education level, RA disease duration, residency (urban vs rural), smoking status, and vaccination status). bDMARDs, biological disease-modifying antirheumatic drugs; csDMARD, conventional synthetic disease-modifying antirheumatic drugs; GC, glucocorticoids; NIRMD, non-inflammatory rheumatic and musculoskeletal diseases, RA, rheumatoid arthritis.

Regarding associations of treatment groups, patients with RA who were only on csDMARDs (HR 1.3; 95% CI 1.1 to 1.5) seemed to have an increased risk compared with patients on bDMARDs or tofacitinib (HR 1.1; 95% CI 0.9 to 1.4) when compared with patients with NIRMD. This risk increased in both csDMARDs (HR 2.7; 95% CI 2.3 to 3.2) and bDMARDs/tofacitinib (HR 2.8; 95% CI 2.2 to 3.5) groups when patients were also on GCs when compared with NIRMD (figure 2). Since tofacitinib is not a bDMARD, we repeated the analyses after excluding patients on tofacitinib (N=304), but the results did not change (data not shown).

## Discussion

In this study following real world patients with rheumatic disease throughout USA, we observed more than 26 000 patients longitudinally with cumulative follow-up of over 100 000 patient-years. Patients with RA had more than a 70% greater risk for SIs compared with those with NIRMDs after adjusting for important confounders. However, the risk attenuated to 30% when further adjusted for GC use. GC use was the strongest contributor to SIs. The most frequent SIs among patients with RA were bacterial infections and those involving the respiratory tract. We found the risk of SI in RA increased in parallel with disease activity and was minimal in those states corresponding to either low disease activity or remission.

The risk of SI in RA compared with a non-RA cohort has been examined in only a few studies to date.^1 28^ Cohort studies describing SIs included very different control groups and were limited due to age restrictions or follow-up period.^13 29 30^ In our study, we examined adults of all ages and leveraged a control group of patients with NIRMD who were followed in the same manner and over the same time period as the patients with RA. Smitten *et al*, in a retrospective claims-based nested case-control study, demonstrated an increased risk of infections in patients with RA (HR 2.0; 95% CI, 1.9 to 2.1) compared with a random sample of patients without RA.^28^ Doran *et al* followed an incident population-based RA cohort where RA cases were matched to non-RA controls. This study reported an HR of 1.8 for SIs leading to hospitalisation in RA compared with non-RA, which is higher than the GC-adjusted HR of 1.3 observed in our study, however similar to the adjusted HR without GC of 1.7.^1^ These studies used the general population or all hospitalised patients as a reference, a difference that likely explains the slightly higher HR compared with our findings that used patients with NIRMD as controls. This is in spite of our broader definition of SIs, which includes outpatient parenteral antibiotic therapy, which is now increasingly used for case finding and has not been consistently captured in prior studies. Having an RA case group followed in an identical manner as the NIRMD group likely reduces selection and reporting bias that may have affected results from other studies. Given that our data set has detailed information on GC use, we can further demonstrate the attenuation of the HR after adjusting for GC which cannot be done in other studies. Furthermore, these earlier studies reported observations from 1955 to 1994 and 1999–2006, respectively, a period when the number and type of disease-modifying RA medications available were limited. The age-adjusted and sex-adjusted HR for SIs in our study was 1.8, which is similar to Doran *et al* where patients with RA had increased risk of infection requiring hospitalisation (HR=1.8; 95% CI 1.5 to 2.2) and compared with the non-RA population. However, we used LASSO, a modern machine learning method, for selecting the best set of variables in the regression analyses, which is important to address confounders.^31^ Thus, our risk estimation for SIs in patients with RA may be less biased than previous studies, nevertheless comparable.

Our study is consistent with studies from different parts of the world where GC use was associated with increased risk of SI in patients with RA.^32 33^ Other studies have suggested that immunosuppressive treatment and bDMARDs further increase the risk of SIs in RA.^34–36^ Our sensitivity analysis showed similar results whereby patients on csDMARDs and patients on bDMARDs or tofacitinib were all at an increased risk of SIs compared with individuals with NIRMD that did not appear to differ meaningfully by DMARD class. The SI risk in patients with RA on csDMARDs and bDMARDs compared with patients with NIRMD was also similar with respect to better control portended lower risk irrespective of treatment. There is also evidence that longer-term use of bDMARDs in the elderly might reduce infection risk by lowering the patient’s disease activity and requirement for GCs, which have consistently been shown to increase SI risk.^30 33^ Our sensitivity analysis showed a dose-dependent relationship between disease activity and SI risk in patients with RA compared with NIRMD. This is consistent with recent literature where lower disease activity was associated with lower SI rate after controlling for potential confounders.^16 37 38^ A recent study which estimated disease activity using multibiomarker disease activity test scores showed similar results that higher disease activity was associated with higher rates of SIs.^39^ While claims-based studies typically lack clinical and patient-reported outcomes, the strength of our study is the availability of comprehensive list of patient demographics, disease activity and severity measures that help to translate our results into clinical practice. These results have a direct application to counsel patients in clinical practice whereby decreasing disease activity helps decrease pain and prevent joint damage and may decrease SI risk which has morbidity and mortality implications.

Recognising SIs as a ‘sentinel outcome’, there have been several attempts to quantify, and predict factors which increase this risk in RA.^40–42^ Some have described aetiologies or site of SIs individually, some have described the risk in regional/hospital cohorts, while others have described increased SIs in a rural population.^29 43^ However, our study is among the first to comprehensively present a national cohort, with both urban and rural representation to characterise the risk associated with SIs with details relevant to aetiology and site. For SIs in RA, we report the most frequent aetiology to be bacterial and most frequent site to be respiratory, findings that are consistent with other studies.^29 44 45^ It is noteworthy that the observed risk of Herpes zoster in our RA cohort against NIRMD was slightly lower than other published studies (HR 1.8 vs 2.4).^46 47^ This may be due to the older NIRMD comparator group (62 vs 58 years for RA) in this study as Herpes zoster risk increases with age. Also, we focused on Herpes zoster SIs rather than non-serious Herpes zoster infections, which led to the lower overall IRs than previous studies.^47^

There were limitations of our study. The vast majority of patients in this cohort were white and female, suggesting that these results may not be generalisable. Since this is a patient-reported longitudinal study, there is likely some participation and recall bias; however, we controlled for this by having an internal comparison group (patients with NIRMD) with similar biases. We may not have been able to capture all SIs as patients who had a serious event may have stopped participating in the study as well as the self-reported nature of the data may lead to patients forgetting to report events; thus, our findings could have underestimated the actual rate of SIs. While it would be interesting to know how individual DMARDs affect the SI risk in RA, it is beyond the scope of this study given that we have used NIRMD as a comparator group to establish the increased SI risk in this study. Further studies detailing the medications would require a different methodological approach where incident medication users are captured and followed for SIs.

Since the SIs were classified on the basis of ICD-9 codes, a number of events did not have a specified site or aetiology of the SIs and were thus labelled ‘unknown’. Similarly, while most SIs had a known site of infection, a significant proportion had an unknown aetiology given that many ICD-9 codes only mention the site and not the aetiology. Also, certain infections such as viral and fungal infections were rare and thus limited study power to detect risk differences.

In conclusion, our study showed an increased risk of SIs in patients with RA compared with patients with NIRMD, and that this excess risk was present for bacterial, respiratory, sepsis, bloodstream, skin, bone and joint SIs. This increased risk can be attributed to both disease and/or its treatment. Use of GCs universally increased the risk of SIs. However, achieving remission or low disease activity state in patients with RA normalised the SI risk against NIRMD. Clinicians should weigh the potential SI risk associated with aggressive treatment strategies in patients with RA while targeting and sustaining remission or low disease activity.
