# Supplementary material for: Serious infection risk in rheumatoid arthritis compared with non-inflammatory rheumatic and musculoskeletal diseases: a US national cohort study
Source: RMD Open. 2019 Jun 9;5(1):e000935. doi: 10.1136/rmdopen-2019-000935 (PMC6560658; doi:10.1136/rmdopen-2019-000935)
Supplement: Supplementary data [file rmdopen-2019-000935supp001.docx]

**Supplementary Material**

**Appendix 1: FORWARD data collection and validation**

FORWARD (formally known as the National Data Bank for Rheumatic Diseases [NDB]) obtains participants primarily by referral from US and Canadian rheumatologists. After preliminary contact and informed consent, staff contact the patient, obtain demographic and follow-up information and a detailed medical history. At 6-month intervals (every January and July), FORWARD participants are surveyed by either a paper, web, or telephone questionnaire.

To validate medical events including serious infections, FORWARD collects medical records for important medical events in addition to confirmatory patient/family inter­views. Most events that result in hospitalization, and medical events deemed important, are validated by obtaining medical confirmation (physician contact or medical record review) after obtaining consent from the participants. In general, FORWARD validates all malignancies, serious infections, non-elective hospitalizations, and medical conditions of interest (e.g., demyelinating disorders). A release for medical records is included in the questionnaire. If a patient reports being hospitalized for one of the above conditions and has signed the release, medical records are obtained from the healthcare provider. The medical records are reviewed and the IDC-9/10 codes and the DRG codes are added to the database. The data in FORWARD uses both ICD-9 and ICD-10. For validated events after the last quarter of 2015 (when ICD-10 were universally used in USA), at a collection level, we converted ICD-10 codes to ICD-9 for comparability.

Outpatient procedures and serious infections are validated in the following way: patients are contacted if they report a biopsy or endoscopy to confirm location of procedure, severity, and diagnosis. If surgery was required hospital records are obtained. Patient will also be called if they have a positive response to 1) septicemia, 2) bone or joint infection, or 3) other lung infections including pneumonia, pneumocystis, histoplasmosis, cytomegalic infection, fungal, or other infections. During the interview, if the patient indicates hospitalization, the data are recorded, and medical records are obtained. Death records are collected for all participants through linkage with the National Death Index. Data collection involves Internet surveys, paper questionnaire scanning, patient interviewing, data extraction from medical records, coding and programming.

All of the validated data (i.e., hospitalizations, outpatient procedures, cancers, strokes, lupus, psoriasis, etc.) contain the following: which questionnaire it was reported on (to link to the patient’s 6-month questionnaire), event date, ICD-9/10 codes, validation source (hospital records, medical records, biopsy repot, physician report, patient report, US National Death Index, family member, or death certificate), hierarchy of the validation (confirmed by medical sources, confirmed by further data obtained from the patient, error/non-event, out of timeframe event), and, for cancers, primary or secondary cancer, cancer status (remission/cured, new, existing, recurrence), and cancer cell type (if applicable).^[[1]](#footnote-1)^

**Table S1 –ICD-9 codes for hospitalized infections and etiology**

| **Outcome: Hospitalized infections** | **Etiology** | **ICD-9-CM code** |
| --- | --- | --- |
| Tuberculosis | Bacterial | 010/011.39, 011.5/018.99 |
| Atypical mycobacterial infections | Bacterial | 031* |
| Cryptococcosis | Fungal | 117.5 |
| Histoplasmosis | Fungal | 115.0/115.99 |
| Aspergillosis | Fungal | 117.3 |
| Coccidioidomycosis | Fungal | 114.0/114.99 |
| Blastomycosis | Fungal | 116 |
| Candidiasis | Fungal | 112.5 |
| Cytomegalovirus | Viral | 078.7 484.1 |
| Viral Hepatitis | Viral | 070/070.9 |
| Hepatitis B | Viral | 070.2/070.33 |
| Hepatitis C | Viral | 070.41, 070.44, 070.51, 070.54, 070.7/070.71 |
| Toxoplasmosis | Fungal | 130/130.9 |
| Pneumocystis carinii | Fungal | 136.3 |
| Human papillomavirus | Viral | 079.4 |
| Herpes simplex | Viral | 054/054.9 |
| Herpes zoster | Viral | 053/053.9 |
| Human immunodeficiency virus (HIV) | Viral | 42 |
| Listeriosis | Bacterial | 010/011.39, 011.5/018.99, 027.0, 117.3, 115.0/115.99, 117.5, 136.3, 112.5,  078.7, 484.1, 114.0/114.99, 054.3, 117.7, 116/116.0, 031* |
| Pneumonia | Bacterial | 481*/483* |
|  | Fungal | 484.6 484.7 |
|  | Viral | 480* 484.1 487 |
|  | Unable | 483.8 584.8 485 486 513 323.8* 323.9 |
| Sepsis/bacteremia | Bacterial | 038*, 995.91, 790.7 |
| Cellulitis | Bacterial | 681/682.9 |
| Meningitis/encephalitis | Bacterial | 320/320.9, 036/036.2, 323/323.9, 049.2, 091.81, 062/063.9, , 094.81, 094.2, 094.81 |
|  | Viral | 54.3 066.4/066.49 |
|  | Unable | 323.8* |
| Endocarditis | Bacterial | 421/421.9, 422.92, 391.2, 036.42, 093.2/093.29, 098.84 |
| Pyelonephritis | Bacterial | 590/590.99 |
| Septic arthritis | Bacterial | 711.0/711.09, 711.9/711.99, 003.23, 098.5/098.59 |
| Osteomyelitis | Bacterial | 730/730.29, 003.24, 376.03 |
| Prosthetic joint infection | unknown | 996.66 |
| Skin | Unknown | 684/686.9 |
| Peritonitis and retroperitoneal abscess or infections | Unknown | 567* |
| Abscess of anal and rectal regions | Unknown | 566 |
| Acute bronchitis and bronchiolitis | Unknown, Respiratory | 466* |
| Liver abscess | Unknown | 572.0 571.1 |
| Acute pelvic inflammatory disease | Unknown | 614.0 614.3 614.5 |
| Orchitis and epididymitis | Unknown | 604* |
| Fasciitis | Unknown | 729.4 |
| Necrotizing fasciitis | Unknown | 728.86 |
| Other | Unknown | 009* |

**Table S2 – Variables selected in each model (including and excluding GC use) by the LASSO selection model and log (lambda), the optimal value associated with the best model**

| **Infection** | **GC use** | **Variables selected by LASSO:** | **Log(lambda)*** |
| --- | --- | --- | --- |
| **All infections** | **Yes** | Age, sex, race, HAQ, pain scale, modified RDCI, education level, RA duration, residency (urban vs rural), glucocorticoid use, prior serious infections, smoking status, diabetic and vaccines | -7.55 |
|  | **No** | Same model without GC | -7.69 |
| **Opportunistic** | **Yes** | Age, sex, HAQ, pain scale, education level, RA duration, glucocorticoid use, prior serious infections, smoking status, diabetic, pulmonary and fracture comorbidities and vaccines | -8.68 |
|  | **No** | Age, sex, education level, RA duration, prior serious infections, smoking status, diabetic, pulmonary and fracture comorbidities and vaccines | -8.46 |
| **Herpes zoster** | **Yes** | Age, HAQ, glucocorticoid use, prior serious infections, smoking status, diabetic and vaccines | -7.95 |
|  | **No** | Race, smoking status, prior serious infections, modified RCDI, HAQ, diabetic, vaccines | -8.27 |
| **By etiology:** |  |  |  |
| Bacterial | Yes | Age, sex, race, HAQ, pain scale, RDCI, education level, RA duration, residency (urban vs rural), glucocorticoid use, prior serious infections, smoking status, diabetic, pulmonary and fracture comorbidities and vaccines | -8.39 |
|  | No | Same model without GC use | -8.45 |
| Viral | Yes | Sex, HAQ, modified RDCI, RA duration, residency (urban vs rural), glucocorticoid use, prior serious infections, pulmonary comorbidities and vaccines | -7.88 |
|  | No | Sex, HAQ, modified RDCI, RA duration, residency (urban vs rural), prior serious infections, pulmonary comorbidities and vaccines | -8.07 |
| Fungal | Yes | Age, sex, race, RA duration, modified RCDI, glucocorticoid use, smoking status, pulmonary and fracture comorbidities and vaccines | -8.59 |
|  | No | Age, sex, race, RA duration, glucocorticoid use, smoking status, pulmonary and fracture comorbidities and vaccines | -8.47 |
| Unknown etiology | Yes | Age, sex, race, HAQ, pain scale, modified RDCI, education level, RA duration, residency (urban vs rural), glucocorticoid use, prior serious infections, smoking status, diabetic and vaccines | -7.93 |
|  | No | Same model without GC use | -8.02 |
| **By site:** |  |  |  |
| Respiratory | Yes | Age, sex, race, HAQ, modified RDCI, education level, RA duration, residency (urban vs rural), glucocorticoid use, prior serious infections, smoking status, diabetic, pulmonary and fracture comorbidities and vaccines | -8.12 |
|  | No | Same model without GC |  |
| Central Nervous System | Yes | Sex, Residency (urban vs rural), glucocorticoid use, prior serious infections, smoking status, and fracture comorbidities | -8.60 |
|  | No | Sex, Residency (urban vs rural), prior serious infections, ever smokers | -8.32 |
| Abdominal | Yes | Age, sex, modified RDCI, pain scale, glucocorticoid use, prior serious infections, smoking status, diabetic and vaccines | -8.02 |
|  | No | Age, sex, RA duration, modified RDCI, pain scale, prior serious infections, smoking status, diabetic and vaccines | -8.45 |
| Urinary | Yes | Age, sex, race, modified RDCI, education level, RA duration, residency (urban vs rural), glucocorticoid use, prior serious infections, smoking status, diabetic, history of fracture and vaccines | -8.19 |
|  | No | Sex, race, modified RDCI, education level, RA duration, residency (urban vs rural), prior serious infections, diabetic and vaccines | -8.01 |
| Bloodstream including sepsis | Yes | Age, sex, race, HAQ, modified RDCI, education level, RA duration, residency (urban vs rural), glucocorticoid use, prior serious infections, smoking status, diabetic, history of fractures, vaccines | -9.03 |
|  | No | Age, sex, race, HAQ, modified RDCI, education level, RA duration, residency (urban vs rural), prior serious infections, smoking status, diabetic, pulmonary and fracture comorbidities and vaccines | -8.84 |
| Skin, bone and joint infections | Yes | Age, sex, race, HAQ, modified RDCI, education level, RA duration, residency (urban vs rural), glucocorticoid use, prior serious infections, smoking status, diabetic, pulmonary and fracture comorbidities and vaccines | -8.66 |
|  | No | Same model without GC use | -8.76 |
| Unknown site | Yes | Sex, race, HAQ, modified RDCI, education level, RA duration, residency (urban vs rural), glucocorticoid use, prior serious infections, smoking status, diabetic, pulmonary and fracture comorbidities and vaccines | -8.38 |
|  | No | Sex, race, HAQ, modified RDCI, RA duration, residency (urban vs rural), glucocorticoid use, prior serious infections, smoking status, diabetic, pulmonary and fracture comorbidities and vaccines | -8.63 |

* Best model corresponds to the minimum value of the cross-validation error curve

*LASSO= Least Absolute Shrinkage and Selection Operator, RA = Rheumatoid Arthritis, HAQ = Health Assessment Questionnaire, RDCI = Rheumatic Disease Comorbidity Index, GC=glucocorticoids.*

**Table S3 -**  **Number of opportunistic serious infections in RA and NIRMD patients**

| **Non-Herpes Zoster Opportunist infections** | **RA** | **NIRMD** |
| --- | --- | --- |
| Tuberculosis | 9 | 2 |
| Candidiasis | 1 | 1 |
| Coccidioidomycosis | 7 | 0 |
| Histoplasmosis | 25 | 1 |
| Blastomycosis | 1 | 0 |
| Aspergillosis | 11 | 1 |
| Cryptococcosis | 5 | 0 |
| Pneumocystis carinii | 8 | 0 |
| Listeriosis | 4 | 0 |
| Atypical mycobacterial infections | 13 | 2 |
| Cytomegalovirus | 1 | 0 |
| Herpes simplex | 0 | 0 |
| Total: | 85 | 7 |

*RA= Rheumatoid arthritis, NIRMD= Non-Inflammatory Rheumatic and Musculoskeletal Diseases*

**Table S4 -**  **Number of infections and Incidence Rates for serious infections in RA patients by disease activity and treatment categories**.

|  | **N** | **Incidence Rates (95 CI%)** |
| --- | --- | --- |
| **NIRMD patients** | 276 | 13.4 (11.9 – 15.0) |
|  |  |  |
| **RA patients** | 1600 | 19.6 (18.7 – 20.6) |
|  |  |  |
| **Disease Activity** |  |  |
| Remission/Low | 604 | 13.4 (12.4 – 14.6) |
| Moderate | 921 | 26.7 (25.1 – 28.5) |
| High | 68 | 41.3 (32.5 – 52.4) |
|  |  |  |
| **Treatment groups** |  |  |
| csDMARDs without GC | 519 | 13.1 (12.0 – 14.2) |
| csDMARDs + GC | 541 | 35.5 (32.5 – 38.5) |
| bDMARDs / Tofacitinib without GC | 88 | 11.5 (9.3 – 14.2) |
| Biologic / Tofacitinib + GC | 88 | 38.1 (30.9 – 47.0) |

*RA= Rheumatoid arthritis, NIRMD= Non-Inflammatory Rheumatic and Musculoskeletal Diseases, csDMARDs= Conventional synthetic disease-modifying antirheumatic drugs, bDMARDs= Biological disease-modifying antirheumatic drugs, GC= glucocorticoid use*

**Figure S1** - Coefficients estimates of the LASSO fit as a function of the regularization term for the outcome all Serious Infections. It can be interpreted as evidence of the variables that enter the model early are the most predictive indication the variables


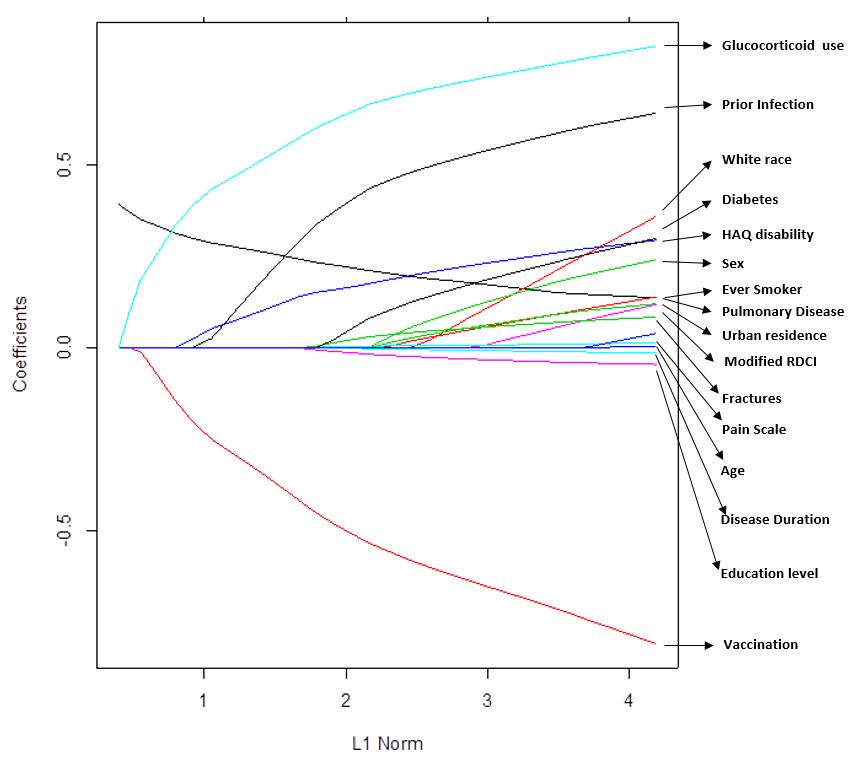


*LASSO- Least Absolute Shrinkage and Selection Operator

*RA= Rheumatoid arthritis, NIRMD= Non-Inflammatory Rheumatic and Musculoskeletal Diseases, RDCI= Rheumatic Disease Comorbidity Index, HAQ= Health Assessment Questionnaire*

**Figure S2** - Cross validation partial likelihood as a function of Log (lambda), the logarithm of the LASSO shrinkage parameter for the outcome All Serious infections for the model including Glucocorticoids.


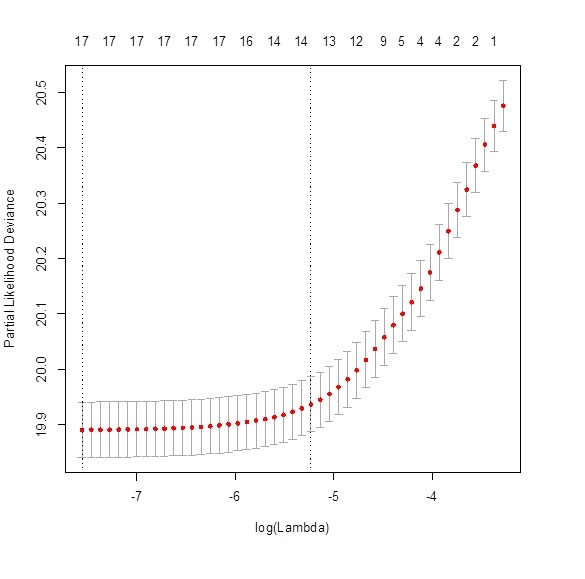


*LASSO- Least Absolute Shrinkage and Selection Operator

1. Wolfe F, Michaud K: **The National Data Bank for rheumatic diseases: a multi-registry rheumatic disease data bank**. *Rheumatology (Oxford)* 2011, **50**(1):16-24. [↑](#footnote-ref-1)
